# Machine Learning for Molecular Modelling in Drug Design

**DOI:** 10.3390/biom9060216

**Published:** 2019-06-04

**Authors:** Pedro J. Ballester

**Affiliations:** Cancer Research Center of Marseille, CRCM, INSERM, Institut Paoli-Calmettes, Aix-Marseille Univ, CNRS, F-13009 Marseille, France; pedro.ballester@inserm.fr

Machine learning (ML) has become a crucial component of early drug discovery. This research area has been fueled by two main factors. The first is the fast-growing availability of relevant experimental data. Examples of such datasets are those containing the bioactivities of molecules of known chemical structure against a non-molecular target (e.g., a cancer cell line), binding affinities of such molecules against a molecular target (e.g., a particular kinase validated for a specific cancer type) or the X-ray crystal structures of a molecular target. This factor has been boosted by the development of community resources, such as ChEMBL [1], PubChem [2], NCI-60 [3], or PDBbind [4], that curate and facilitate re-using these datasets for predictive modelling. The second factor is the easy access to high-quality and well-documented implementations of a range of ML algorithms, including those of recent advances such as XGBoost [5], deep learning [6], or conformal prediction [7]. As a result, an increasing number of data-driven ML models have been proposed and found advantageous in some way in identifying new starting points for the drug discovery process.

This Special Issue showcases five studies investigating the application of ML for molecular modelling in drug design. These studies have been carried out by 21 academic and industry researchers from around the World. ML techniques include support vector machines (SVM), random forest (RF), *k*-nearest neighbors (*k*-NN), convolutional neural network (CNN), or recurrent neural network (RNN), either alone or integrated with dimensionality reduction techniques such as GA(genetic algorithm)-based feature selection (FS) and principal component analysis (PCA).

The first of these papers by Cruz et al. [8] investigated quantitative structure–activity relationship (QSAR) models to predict which molecules are able to inhibit the growth of HCT116, a human colon carcinoma cell line. Regression models were developed with this purpose, using a total of 7339 molecules with chemical structure and half-maximal inhibitory concentration (IC_50_) data. The QSAR classification models were also built, this time using nuclear magnetic resonance (NMR) data as features. Models were built with *k*-NN, RF, and SVM algorithms. The authors concluded that the developed models were sufficiently predictive to permit the identification of new inhibitors of this non-molecular target.

Chen et al. [9] aimed at identifying new inhibitors of the C1 target that could be used to advance towards new treatments for hereditary angioedema. The QSAR models were built integrating SVM with PCA and GA-based FS. Once these models were retrospectively validated, they were used to screen 72 million PubChem compounds against C1. Large hit rates were obtained following in vitro tests. Some of these new inhibitors have previously unknown active scaffolds for this target and are single-digit μM.

Detection of mutagenicity during early stages of drug discovery is important to reduce the likelihood of developing drugs with harmful side effects. Norinder et al. [10] applied the conformal prediction method to the prediction of mutagenicity of primary aromatic amines (PAAs) using Leadscope features in conjunction with RF. Conformal prediction is attractive in that it predicts how reliable model predictions are. Such RF-based QSAR models were built and validated. The authors concluded that it was possible to predict this type of mutagenicity in an independent set of compounds while estimating the errors of each of these individual predictions using their methodology.

Bjerrum and Sattarov [11] demonstrated that the QSAR model accuracy can be improved by using heteroencoders of the molecules as features. The common approach of using autoencoders on canonical simplified molecular-input line-entry system (SMILES) is hampered by their poor neighborhood behavior (i.e., similar chemical structures mapping onto dissimilar canonical SMILES). A heteroencoder is introduced as an autoencoder considering several non-canonical SMILES as input, instead of a single canonical SMILES, for each molecule to factor in the impact of different chemical representations on modelling. These heteroencoders were trained using CNNs and RNNs with long short-term memory cells. In comparison to using autoencoders, the use of heteroencoders resulted in better predictive performance of the resulting QSAR models. Furthermore, the spanned latent space led to a better agreement between SMILES similarity and circular fingerprint similarity of the considered molecules.

Machine learning has been used to generate diverse ligand-based predictive models in these four contributions so far [8,9,10,11] by exploiting chemical structure and bioactivity data. However, by also exploiting X-ray crystal structure data, ML can also be used to build protein-ligand predictive models. These models are known as ML scoring functions (SFs) and have been found to be an important complement to classical SFs in docking [12]. The last paper in this issue [13] investigated whether the well-known superiority of ML SFs over classical SFs on average across targets is exclusively due to the presence of training complexes with highly similar proteins to those in the test set. We addressed this question by using 24 similarity-based training sets, a widely used test set, and four SFs. We found that an RF-based SF outperforms the best classical SF even when 68% of the most similar proteins are removed from the training set. In addition, unlike the classical SF, the RF-based SF is able to keep learning as the training set size grows, becoming substantially more predictive when the full 1105 data instances are used for training. These results show that ML SFs owe a substantial part of their performance to training on complexes with dissimilar proteins to those in the test set.

## References

[1] Bento A.P., Gaulton A., Hersey A., Bellis L.J., Chambers J., Davies M., Krüger F.A., Light Y., Mak L., McGlinchey S. (2014). The ChEMBL bioactivity database: An update. Nucleic Acids Res..

[2] Wang Y., Suzek T., Zhang J., Wang J., He S., Cheng T., Shoemaker B.A., Gindulyte A., Bryant S.H. (2014). PubChem BioAssay: 2014 update. Nucleic Acids Res..

[3] Shoemaker R. H. (2006). The NCI60 human tumour cell line anticancer drug screen. Nat. Rev. Cancer.

[4] Li Y., Han L., Liu Z., Wang R. (2014). Comparative assessment of scoring functions on an updated benchmark: 2. Evaluation methods and general results. J. Chem. Inf. Model..

[5] Sheridan R.P., Wang W.M., Liaw A., Ma J., Gifford E.M. (2016). Extreme Gradient Boosting as a Method for Quantitative Structure-Activity Relationships. J. Chem. Inf. Model..

[6] Ma J., Sheridan R. P., Liaw A., Dahl G., Svetnik V. (2015). Deep Neural Nets as a Method for Quantitative Structure-Activity Relationships. J. Chem. Inf. Model..

[7] Norinder U., Carlsson L., Boyer S., Eklund M. (2014). Introducing Conformal Prediction in Predictive Modeling. A Transparent and Flexible Alternative to Applicability Domain Determination. J. Chem. Inf. Model..

[8] Cruz S., Gomes S., Borralho P., Rodrigues C., Gaudêncio S., Pereira F., Cruz S., Gomes S.E., Borralho P.M., Rodrigues C.M.P. (2018). In Silico HCT116 Human Colon Cancer Cell-Based Models En Route to the Discovery of Lead-Like Anticancer Drugs. Biomolecules.

[9] Chen J., Schmucker L., Visco D., Chen J.J., Schmucker L.N., Visco D.P. (2018). Pharmaceutical Machine Learning: Virtual High-Throughput Screens Identifying Promising and Economical Small Molecule Inhibitors of Complement Factor C1s. Biomolecules.

[10] Norinder U., Myatt G., Ahlberg E., Norinder U., Myatt G., Ahlberg E. (2018). Predicting Aromatic Amine Mutagenicity with Confidence: A Case Study Using Conformal Prediction. Biomolecules.

[11] Bjerrum E., Sattarov B., Bjerrum E.J., Sattarov B. (2018). Improving Chemical Autoencoder Latent Space and Molecular De Novo Generation Diversity with Heteroencoders. Biomolecules.

[12] Ain Q.U., Aleksandrova A., Roessler F.D., Ballester P.J. (2015). Machine-learning scoring functions to improve structure-based binding affinity prediction and virtual screening. Wiley Interdiscip. Rev. Comput. Mol. Sci..

[13] Li H., Peng J., Leung Y., Leung K.-S.K.-S., Wong M.-H.M.-H., Lu G., Ballester P.J.P. (2018). The Impact of Protein Structure and Sequence Similarity on the Accuracy of Machine-Learning Scoring Functions for Binding Affinity Prediction. Biomolecules.

